# Selections and Abstracts

**Published:** 1902-04

**Authors:** 


### *SELECTIONS AND ABSTRACTS.*

### Cerebral Hemorrhage.

This important topic is the subject of discussion in a paper by Dr. William Browning in the *New York Medical Journal* for February 15. After calling attention to the measures to be adopted for prophylaxis he emphasizes the importance of administering as quickly as possible after the onset of the attack, since this is "emergency work," powerful and quickly acting vasodepressants. The favorite remedies are gelsemium, aconite and veratrum. Dr. Browning prefers the first of these, commencing with a 10-drop dose of the fluid extract and continuing with 5 to 10-drop doses as indicated. Gelsemin may be preferred, as being more convenient and exact ; the dose at first is 1-10 to 1-8 grain, followed by 1-20 grain doses. Aconitin is also efficient in 1-100 to 1-50 grain doses. It is important the initial doses of these depressant drugs be large or that they be given at short intervals, in order to get the physiological effects as soon as possible.

Dr. Browning also places due emphasis upon immobilization of the patient in the semi-reclining posture, immediately, wherever he may be. "When surroundings permit, the patient should be cared for at the place where seized. I have frequently pre-empted a parlor, a dining-room, or a library. An embargo should be promptly placed on any interruption, noise, or disturbance. A set of cushions or a cot mattress can be secured almost anywhere, and the patient made somewhat comfortable, even on the floor. Transportation must, if possible, be postponed until we feel confident that it is permissible. We cannot guard too carefully against an immediate recurrence from the same vessel."

He speaks as follows of other therapeutic measures:

"Purgatives may be needed; yet, after all, it is not well to have our patient disturbed by a bowel movement while the ruptured vessel is still bleeding. In this respect, also, present teaching should be modified to favor temporary immobilization. Calomel, as it is somewhat of an intestinal antiseptic, rather soothing to the stomach, and not hasty in action, can most safely be given.

"Phlebotomy is more than replaced by the depressants. Position is of importance—viz., reclining to an extent that fully relaxes the muscles and yet does not depress the head, and for most persons a resting on the right side. When compelled to use quieting remedies give bromid or a coal-tar product, but never any opiate.

"The use of gelatin internally, either subcutaneously or possibly by the mouth, seems to be attracting attention for its hemostatic qualities in all concealed hemorrhages. It acts simply by increasing the coagulability of the blood. For injection in the human being, 250 cubic centimeters of a 1 per cent. solution in normal saline fluid is recommended. By the mouth one observer has given 200 cubic sentimeters of a 10 per cent. solution daily, though there can hardly be any necessity for fixing such a narrow limit. Although a method of some promise for cerebral cases, it must be slower in action than by depressants."

For special symptoms the following are suggested:

"For the headache that so frequently attends the attack, antipyrin or its allies in small doses does well, is also rather soothing to the stomach, and is the safest agent where plain bromids fail. Much the same applies to the special restlessness that often threatens to harm as much as any muscular effort. Still, for this, the depressants—by controlling the main process—act best. Convulsions are rare, but may call for the administration of a few whiffs of chloroform; and catheterism may be necessary to empty the bladder.

"Dont's.—Don't give stimulants. Their use in such cases is most reprehensible. So often we see them freely given, notably the alcoholic. The patient is prostrated, and the lay mind naturally turns to tonics and bracers—about the worst thing that can be done.

"Don't resort to saline injections. During the acute stage a limitation of fluids is in order.

"Don't use the depressant diaphoretics, such as ipecac, pilocarpin or apomorphin. They tend to nauseate, an inclination otherwise too common, and, in the degree of attempts at vomiting, most undesirable.

"Don't prescribe digitalis. I have repeatedly seen it bring on another attack. It is a dangerous drug in any individual with a liability to apoplexy, and for this, if no other reason, of questionable utility in nephritics. Where anything of the sort must be used, strophanthus is in my experience far safer.

"Don't resort to opiates. They are likewise contraindicated.

"Don't try nitrates, as their use in any form is here out of place.

"Don't permit any muscular exertion on the patient's part; and moving by others should be limited as much as possible."

In about a week, if the patient shows improvement, slight exercise is allowed and the patient is encouraged to sit up so as to stimulate the physical and mental faculties. When there is chronic paralysis the author advises teaching the patient to use the affected member, little by little.—*Medical Standard.*

---

### THE SOIL AND SEED OF TUBERCULOSIS.

In the January issue of the *Journal of Tuberculosis*, Dr. H. E. Lewis emphasizes his belief that the development of tuberculosis in the individual is the result of a coincidence of not one, but of several conditions. Those conditions are: First, a potent tuberculous infection depending for its potency on a certain degree of virulence; second, a certain negative chemic or histologic condition of the lymph nodes, resulting from hereditary tendencies or from circumstances of environment, which fails to arrest or inhibit the growth and systemic ingress of potent tubercle bacilli; third, a retrograde metamorphosis of structural cells in some part of the body (more particularly in the lung), from trophic, traumatic or toxic influence which favors the growth of the invading germ.

The first two conditions, of course, are relative, that is, a particularly large amount or a specially virulent tuberculous infection might overcome resistant conditions of the lymphatic system that ordinarily would be effective against a less virulent or smaller amount of ineffective material. And conversely a weakened lymphatic system might prove vulnerable to what under ordinary conditions would be non-potent tuberculous material. In other words, the success of tubercle bacilli in making a systemic ingress depends upon bacterial power, toxic or vegetative, which is numerically or through special potency greater than the forces, chemic or histologic, of the lymph nodes which tend to resist or prevent such ingress.

Reduced, then, to its lowest terms, the question is one of soil and seed, but, though the phrase is a trite one, the author, in the light of his own experiments, is enabled to give it new interest. As to soil, the writer does not ascribe as much influence to heredity as it is commonly credited with. In 176 cases he found tuberculosis, positive or suspected, in the parent or grandparents of only 42. He is inclined to think that a tuberculous ancestry has no more causative bearing on the production of a fertile soil than many other chronic ailments tending to histologic vitiation.

As to seed, the writer notes that there is abundant proof to indicate that tubercle bacilli show variation in pathogenic intensity. Theobald Smith has shown that bacilli from the cow, horse, and man differ in degree of virulence. Arloing and Lingard have demonstrated that tuberculous material from lesions in the lungs kills guinea pigs much quicker than that taken from caseous glands in other parts of the body. The author has proved that infective material from widely different types of pulmonary tuberculosis shows marked variations in virulence for the lower animals. The experiments were in part as follows:

One group of five guinea pigs and four rabbits, after aseptic preparation, were inoculated with sputum from two cases of rapidly fatal acute pneumonic phthisis; and a second group with sputum from two chronic cases, both of over one year's duration, but with slight invasion of the lung substance. Of the first group, the guinea pigs all showed extensive lesions in five to fourteen days, and all but one died inside of eight weeks. All of the rabbits also showed comparatively rapid extension of the tuberculous infection, but the results in the second group were quite different. In the same number of animals the extension of the disease from the inoculated area was delayed two or three times as long, and only one of the guinea pigs died within three months. Of the second group of rabbits only two gave any reaction to the inoculations whatsoever.

Certain pseudo-tubercular conditions are also of interest. Flexner has described a caseous pneumonia and a nodular condition of the peritoneum produced by a streptothrix. The fact that small branched forms of tubercle bacilli are occasionally found in tuberculous lesions resembling the actinomyces, to which the above streptothrix probably belongs, may show that these pseudo-forms of tuberculosis bear some relation to the real disease after all.

Moller describes a grass bacillus, which he found in barn-dust, and which is pathogenic to guinea pigs, producing lesions almost identical microscopically with those of tuberculosis. The author reports a bronchitis in a dairyman, clinically resembling a tuberculous infection, but in which the writer thinks that a species of hay bacillus was the offending micro-organism. Marzinowsky, Fraenkel and others have found other bacilli which have the morphologic and staining peculiarities of tubercle bacilli.

From this it seems that there are substantial grounds for the belief that the tubercle bacillus is evolutionary in character, which evolution is evidenced by certain variations and gradations in a morphology and virulence modified by the chemic or biochemic conditions of different environments.—*St. Louis Med. Review.*

---

### THE MODERN THERAPY OF GONORRHEA IN THE MALE.

Since the discovery of the gonococcus by Neisser, nearly two decades ago, much has been done to simplify the treatment of this disease, so that to-day we have a few methods which if followed with care will ultimately bring us to a cure in this troublesome affection.

Upon the presentation of a case of urethritis the first thing that the physician has to find out is whether or not he has to deal with a case of specific or non-specific urethritis, and whether the disease is affecting the anterior or posterior urethra or both. In order to determine if the diplococcus be present or not, recourse must be had to the microscope, and it might be said in passing that it is impossible to treat intelligently a gonorrhea without the aid of this instrument of precision.

To prepare the specimen for examination requires but a few minutes. Smear the slide with a small drop of the pus, pass it through a spirit lamp or Bunsen burner, drop on a few drops of methylene blue, wash under the tap, dry with filter-paper, place a drop of cedar oil upon the now stained preparation, and examine with a one-twelfth immersion lens. If the diplococcus be present it may easily be distinguished by its characteristic bean-shaped appearance and grouping. This test is usually sufficient when accompanied by the usual clinical symptoms. In case of doubt recourse must be had to the Gram method. In this the slide is smeared and fixed as just described, and a few drops of anilin-gentian-violet are dropped upon the smear and allowed to remain from one to two minutes. Remove the surplus solution with filter-paper, but do not wash with water. Next immerse in the Gram solution for a similar period. Decolorize with absolute alcohol until the specimen is clear to the naked eye. This should take about one and a half minutes. Now wash in water (the Gram test being finished) to remove the alcohol, and restain with a solution of Bismarck brown. If this test has been carefully carried out the gonococci will be found to have taken the Bismarck brown or secondary stain, while the pseudo-cocci retain the Gram color. Having satisfied himself as to existence of the gonococcus the physician next proceeds to find out if he has an anterior or posterior infection to deal with. This can easily be determined by the Thompson two-glass test.

A marked change has taken place in the treatment of this disease since the introduction of preparations which are not precipitated by albumen. These act purely as disinfectants, and have no irritating or astringent effect on the mucous membrane of the urethral canal.

In dealing locally with an anterior specific urethritis, the drugs available for its treatment may be divided into three groups: (1) simple disinfectants, such as nargol, protargol, and largin; (2) antiseptic astringents—argentamin and nitrate of silver; (3) simple astringents—zinc, sulphate, permanganate of potassium, and alum.

In the acute stage, in the opinion of the writer, the first group will be found to be the most effective, as at this time the gonococci are deep down in the epithelial layer and must be reached to be destroyed.

Nargol meets this requirement best of any drug which we have at present. It is a chemical compound of silver with a protein base. At the commencement of treatment a $\frac{1}{4}$-per-cent solution should be injected into the uretha every eight hours, and the injection to be effective should be retained for from five to ten minutes. It will be found that if this procedure be carefully carried out, within three or four days there will be a marked diminution of the discharge, and the scalding on urination will be markedly lessened. At this time the strength of the nargol solution may be increased to one per cent or even more, depending upon the susceptibility of the individual. Largin will also, on account of its combined bactericidal and astringent powers, be found of value, but its use is often attended with more or less pain, owing to its having a slightly alkaline reaction. If, however, it can be borne, its use is to be recommended, owing to its having a greater penetrating power, its action on the germs being therefore more energetic. This treatment will usually shorten the acute stage to about ten days, and it will be then possible to alternate the disinfectant with the astringent.

The various negative results published of the effect of the silver compounds are undoubtedly due to the changes in the solution. The following method for preparing the injections will, if followed, be productive of the best results: The powder must be dissolved in cold water. An open vessel containing water is stood in a cool place, the powder is dusted on to the surface and allowed spontaneously to be dissolved, and subsequently sufficient cold water is added, to dilute the solution to the required strength. The solution should be freshly made, for stock solutions tend to change color, which is an indication of the splitting up of the organic silver compound. The addition of glycerin to the solution is not a good practice, as it can only act as an irritant.

At this time an examination with the microscope will reveal the fact that while we have not as yet entirely destroyed all the gonococci the number has been considerably lessened. Owing to the peculiar histological structure of the urethra, containing as it does so many crypts and lacunæ wherein the germs may be located, these remedies are by no means specifics, and at present it is impossible to shorten the duration of the disease much under six weeks. The irrigation method of treatment is one which in the hands of many has not proven very successful. This may or may not be due to faulty technique. Irrigations with hot solutions, whether of permanganate of potassium, normal salt solution, or boric acid, are certainly beneficial if properly carried out. They require time, however, and very few patients are willing to appear at the physician's office twice or three times a day for them.

In conjunction with the local treatment already described, attention must, of course, be given to the patient's diet; a lowered one must be enjoined, together with a total abstinence from all liquors. As to internal treatment, the drug of most value is oil of sandalwood administered in ten-minim doses every four hours.

In quite a large proportion of cases of gonorrhea, infection of the prostate is a consequence. When the condition is present our attention must be given to this gland, which must be carefully massaged at frequent intervals. The most important factor in the finishing of a case of gonorrhea is the cold steel sound; in fact, without this instrument, in cases of long standing, it will be impossible to reach a successful termination. The sounds most usually used are those numbered from 12 to 19 American. The sounds should be clean, smooth, and perfectly nickel-plated. Sounds should be allowed to remain in the urethra for five minutes before being withdrawn. They are first applied once a week, then once in two weeks, and finally once a month. The pressure on the tissues made by the steel sounds causes the absorption of the remains of the infiltrated elements of the mucosa, and also maintains unaltered the caliber of the urethra.

Of all the diseases which a physician is called upon to treat, none resist treatment so much as cases of gonorrhea. Ricord is credited with making the remark some years ago that "man knows when gonorrhea commences, but God alone knows when it ends." The only way that we can be reasonably certain that gonorrhea is ended is by a careful microscopical examination of the shreds found in the urine of a gonorrheal patient. If atter several examinations of these shreds no gonococci are found, then may we pronounce the individual cured. This is a question of vast importance and demands the greatest of attention, involving as it does the welfare of many an innocent woman. Many women after marriage begin to suffer from troubles hitherto unknown to them. They recognize, as Julien says, the origin of all their trouble as the wedding trip. Noeggerath has claimed that latent gonorrhea in man will produce latent gonorrhea in the female, which in the course of time will manifest itself by perimetritis and oöphoritis. Finger, before allowing a man suffering from gonorrhea to marry, requires three conditions, viz.: an absence of the gonococci, of pus corpuscles, and of periurethral complications.

Regarding the time that should elapse after the disappearance of the gonococci before a patient should be allowed to marry, it is best to wait until six months have elapsed to be certain that the mucous membrane of the urethra is again normal, or as nearly so as it is possible to make it.—*Wm. C. Martin, M.D., Detroit, Mich.*, *in Medical Age.*

---

### THE "SEPTIC CLAUSE" IN ACCIDENT POLICIES.

What constitutes septic poisoning, according to the definition given by some of the insurance companies, would indicate that there is surely a protection for the company. But what about the insured? If there be a loophole it is surely on the side of the company. I am convinced that several physicians, with whom I am acquainted, are under the impression that if sepsis results after a surgical operation, and the abrasion existed previous to the operation, their policy protects them. It may be well for every physician to inspect his policy and perhaps, if it be not clear, ask the surgeon of the company to define its meaning. Quite frequently a wound is so trivial that it is unnoticed by a surgeon; septic matter finds an entrance and he is disabled. Under these circumstances but few companies offer protection. Yet this is not the impression that prevails among many physicians. It might be well for us to refer to our policies and read the "Septic Clause" carefully, and not wait until disability occurs. Our policy might be weighed in the balance and found wanting. The time to repair a roof is before the rain; procrastination is an inexcusable error.

Dr. G. W. Kemper, of Muncie, details his experience in the *Philadelphia Medical Journal*, February 15, 1902. He says:

"On May 3, 1901, I assisted my son in amputating the thigh of a girl suffering from sarcoma of the knee-joint. After the operation was completed, I took the amputated limb out of doors, and made several incisions into the diseased structures—the knee-joint being in a foul condition—in order to observe the extent of the disease. For some months prior to this event I had been troubled with a slight eczematous eruption at the top of my right ear, and at the close, while washing my hands, to relieve the itching, I accidentally rubbed my affected ear. On May 7th, four days after the operation, a swelling began on my right ear, and developed on the following day a case of erysipelas which extended over my face and scalp. I was confined to my bed and room for three weeks. During the second week, two small abscesses formed in the upper lid of my right eye, and a third one beneath my left eye—all necessitating lancing. After restoration to health, I sent a report of my condition to my company, and received the following reply:

"'Your policy does not cover diseases of any kind. On the contrary, diseases are expressly excepted from the operation thereof. This company, we may say, does issue policies covering the disease from which you were suffering, and we regret for your sake that you had not one of these policies, as you would then be entitled to have made a claim for indemnity against this company.'"

Dr. Kemper wrote to several companies for an opinion in reference to sepsis, as defined in their respective policies, and there was no material difference. The unique answer of Dr. C. W. Hitchcock, Chief Surgeon of the Standard Life and Accident, is as follows:

"We do not intend to cover those cases of sepsis which are so frequent, and which are purely due to the physician's own carelessness, where he has a wound, or other abrasion on his hand or finger and carelessly opens abscesses or dresses purulent or septic cases without taking any adequate precautions to protect his open wounds. Although the surgeon is the last one who ought to leave such wounds unprotected, it is surprising how often cases of this kind of sepsis arise, and we have worded our septic clause as we do because such sepsis is not purely accidental, but is the natural result of the physician's own carelessness, and for this the Accident Company surely should not suffer. I think our septic clause is a fair one and that it concisely expresses just what it is intended to cover."

Dr. Kemper says further :

"The Casualty Company in which I am insured does not stipulate that infection must be received through a recent wound. It carries a very liberal construction of septic poisoning, and implies that it covers any and all forms of 'septic poisoning accidentally incurred.' For this reason I felt that I was justly entitled to the stipulated indemnity.

"The company claimed that I simply suffered from erysipelas, that it did not arise from infection, and did not come under the definition of 'septic poisoning.' They claim, in short, as stated, that erysipelas is a disease and not a product of sepsis, and therefore rejected my claim.

"I feel that I should not allow Dr. Hitchcock's statement to pass unchallenged, when he asserts that the physician is careless who contracts infection through an abrasion or wound on his hand or finger. It is no uncommon thing for a surgeon to become infected through so simple an avenue as a hang-nail upon his finger. A surgeon who is aware of a sore upon his finger should, undoubtedly, guard against danger in operating upon diseased structures, but he may have a broken surface of the skin and not be aware of it. A very minute wound often becomes infected. A surgeon, in cleaning his hands with a nail-brush may cause a slight break of the cuticle, which will become a gateway for infection. It is an awkward matter for a surgeon to cut his hand with a knife, or pick his finger with a needle in operating, and yet at times it seems to be unavoidable. Practically, the surgeon who cuts himself with a knife, or picks himself with a needle, is as careless as the surgeon who neglects to protect a broken surface. Neither should be censured in harsh terms. Between the two accidents there is but slight difference, really none as to results, and yet insurance companies pay for the one accident, and refuse to pay for the other!

"In summing up, I think we may declare the following propositions:

"1. That the surgeon who is insured and receives a disabling wound unfitting him for practicing his profession, is entitled to an indemnity whether he contracts septic poisoning or not.

"2. A surgeon, while operating, may become infected through an old injury, or a new wound. The effects are the same in either instance. There is no valid reason why a policy should not indemnify alike in both cases.

"3. If a surgeon cannot recover indemnity from an infection received through a sore, or an abrasion, then he gains nothing from a clause or rider attached to his general policy. In other words, the term 'septic poisoning' in an accident policy is simply an aid to secure policy-holders!"—*Med. and Surg. Monitor.*

---

### ADENOIDS OF THE PHARYNX.*

Gentlemen of the Society: I will call your attention this evening to adenoids. I will deal with the subject very briefly; I shall not give a pathological description of this malady, but simply call your attention to this unfortunate condition, which is opportune at this particular season of the year. Many of our writers consider adenoids the result of frequent inflammatory processes of the upper air passages, while other equally eminent writers believe that heredity plays an important part in the production of these troublesome growths. Our attention was called to these lymphatic hypertrophies about forty years ago; only in the last ten years

*Read by P. Richard Taylor, M. D., Dean of the Hospital College of Medicine; Professor of Clinical Ophthamology; Professor of Otology and Laryngology, etc., before the Falls City Medical Society, January, 1902.

have they received anything like the attention due them. This abnormal tissue is found in the vault on both sides of the posterior wall of the pharynx and oftentimes throughout the entire pharyngeal mucous membrane. The peculiar facial expression of children affected with adenoids, associated with deafness, generally leads us to suspect that these hypertrophies exist in the pharynx. In extreme cases there is difficulty in eating and swallowing, and in very small children interference with nursing; a discharge from the nose more or less profuse. These children, as a rule, are weak, delicate, and poorly nourished, generally dull and listless, and at all times stupid. With all these symptoms present, a diagnosis of adenoids can safely be made, but with the pharyngeal mirror or the finger all doubt of their presence can be eliminated. From the time that our attention was first called to adenoids, in 1860, up to a few years ago, it was supposed that these growths would disappear about the age of puberty, but clinical experience has taught us that this is the exception and not the rule, for we frequently remove adenoids as late as the age of forty which have existed since childhood.

Most of the distressing symptoms disappear about the age of puberty, from the development and widening of the pharynx at this time, making the obstruction and interference by these growths proportionately less. The nutrition of the child usually improves; the embarrassment to the respiration is lessened. The facial expression changes, and oftentimes all the distressing symptoms disappear except the peculiar nasal voice and the deafness, which persist in almost all cases.

Operative interference is necessary in all cases to give the desired relief. Internal medication and topical application alone have failed to give good results. Adenoids should be operated on as soon as diagnosed. The denser growths should be removed with forceps and curettes, while the superficial ones may oftentimes be removed with the curette alone. If the operation be thoroughly done there is no likelihood of the return of the growth. Secondary operations are usually necessary where the former operation was incomplete.

Whenever adenoid tissue is left in the pharynx it will always continue to offend. General anesthesia is to be avoided whenever possible; if this be unavoidable, the patient should be completely anesthetized and the operation done with the patient's head turned slightly to one side and so low that the blood will flow from the nose and mouth instead of being dammed back upon the larynx. I believe that most of the deaths which occur during this operation under general anesthesia are due to the blood running into the larynx. Chloroform I believe to be the safest anesthetic to be used in the removal of adenoids.

A thorough operation will always remove the peculiar facial expression, the embarrassment to the patient's respiration, and the nasal sound in the voice. The improvement in hearing is usually very marked in the majority of cases, but oftentimes the adenoids extend well up the Eustachian tube, where they cannot be reached with the forceps or curette, and deafness in these cases will persist. Seventy-two per cent. of the cases of deafness are due to adenoids. The earlier the diagnosis and the earlier the case is operated on the better will be the result. We are led to believe that adenoids are oftentimes congenital. Dr. Walker, of New York, reports a case operated on at the age of eighteen days. Many other cases are reported where the operations are done on children only a few months old. A failure of the practitioner to recognize this condition and to recommend its removal will always result disastrously to his patient.—*American Practitioner and News.*

---

### PROLONGATION OF PREGNANCY.

Many years ago (1816) Sir Charles Clark, when giving evidence before the House of Commons, said that he had never yet seen a single instance in which the laws of nature had been changed, believing the law of nature to be that parturition should take place forty weeks after conception; but Dame Nature frequently avails herself of a feminine prerogative and becomes at times fickle and erratic. Many physiologic functions, such as dentition, puberty, or menstruation vary as to the time of occurrence. We have recently seen a case in the practice of a Philadelphia physician in which a child was born with two well-developed teeth in the lower jaw. Louis XIV., le Grand Monarque, was accredited with having the same number at his birth, and Haller had collected nineteen cases of children born with teeth. So many times Nature delights in surprising us with the curious and anomalous. Prolonged pregnancies are interesting from the medico-legal, as well as the scientific, standpoint. Although the normal duration of pregnancy is about 275 days from the cessation of the last menstrual period, yet reliable observers have reported instances in which the time was prolonged much beyond this. Taussig (*Amer. Jour. Obstet.*, October, 1901), has collected 61 cases of partus serotinus which are well authenticated, and he quotes from Issmer some interesting conclusions in regard to the influences which tend to prolong gestation. Issmer finds that the duration of pregnancy increases with each child until the ninth, and then there is again a decrease. The age of the mother is also an important factor, as every pregnancy up to the thirty-fifth year of the mother's life is four or five days longer than the previous one. The social condition plays a part, as it has been found by Penard that of 1,000 pregnancies among working women, 51 per cent. were concluded before 280 days had elapsed, whereas of 1,000 women without active occupation, only 34 per cent. were delivered before 280 days. These figures show the influence of rest upon the lengthening of pregnancy. Women who have been vaginally examined are on an average confined 5.2 days sooner than those not examined. Issmer has also found that the average duration of pregnancy in 912 strong women was 278.6 days; in 288 weak ones, 276.8 days. In Taussig's patient, labor began 323 days after the beginning of her last menstruation. The longest pregnancy recited by him was reported by Puppe, the child being encephalic, and the estimated duration being 348 days. From the medico-legal standpoint, very contradictory evidence has been given by distinguished obstetricians. In the United States, authorities have generally upheld the view that gestation may be prolonged, and it has been judicially decided that it may last 317 days. The period of gestation is frequently a matter of judicial inquiry, particularly in bastardy proceedings, and in controversy among heirs affecting legitimacy; but, as Baker, in a presentation of this subject from a legal standpoint has said,

"The light of the courts in this matter is reflected light, and physicians must determine the matter; and if the space between the maximum and minimum periods hitherto allowed is shown to be too long or too short, the courts will readily follow the truth as it is made manifest." The civil code of France provides that 300 days shall constitute the longest period of the legitimacy of an infant; the Scottish law 300 days; the Prussian 301 days. However apocryphal some of the cases reported may be, yet it is probably true that in about 6 per cent. of pregnant women, the duration of pregnancy is over 300 days; and Von Winckel's statistics show that prolongation occurs in 11 per cent. from 302 to 322 days. In view of the fact that the worst instances of dystocia occur in such cases, it is probably a good rule never to permit pregnancy to continue more than two weeks beyond the normal limit, as the careful induction of labor will be less dangerous than a further continuance of the state of pregnancy.—*American Medicine.*

---

### GONORRHEAL VULVOVAGINITIS IN YOUNG CHILDREN.

Peterson summarizes as follows:

1. Vulvovaginitis in the young girl may be divided into simple and gonorrheal.

2. Simple catarrhal vaginitis is due, in a large majority of cases, to lack of cleanliness, and subsides when the proper treatment is instituted.

3. Gonorrheal vulvovaginitis in young children is more common than is generally supposed. While more frequently met with amidst unhygienic surroundings in large cities, it is by no means a rarity in the less thickly settled districts.

4. Gonorrheal disease is more frequent below the age of six, and it is more common in girls than in boys.

5. Specific vulvovaginitis in the large majority of cases arises from actual contact of the patient with some infected person. A study of the reported epidemics, however, shows that the disease may be spread by other means, such as common bath, towels, bed-linen, etc.

6. The ordinary staining methods will prove satisfactory in making a differential diagnosis between specific and other forms of vulvovaginitis.

7. The parts affected in their order of frequency are the labia, urethra, vagina and cervix; the vagina is more frequently affected in the child than in the adult, owing to the character of its epithelium.

8. The tubes, ovaries and peritoneum may be involved in the pathologic process. It is not improbable that certain diseases of adult life may be ascribed to gonorrheal infection in infancy.

9. Purulent ophthalmia and rheumatism are quite frequent complications. The strictest prophylaxis should be observed in order to avoid the former.

10. The treatment of specific vulvovaginitis must be energetic to be of any avail. Under certain conditions the vaginal orifice should be widely dilated and the vaginal pus cavity properly drained.

In order to abate or prevent a recurrence of the redness and edema of the vulva and vestibule these parts must be kept clean by constant washings with sterilized water or a 5 per cent. solution of boracic acid. The parts should not be wiped, but should be douched off as frequently as may be required. The child will strenuously object to this, or, in fact, any mode of treatment, but the mother must be made to understand the necessity of carrying out the physician's orders if the disease is to be got under control.

In many cases the vaginitis is continued by the damming back of the purulent secretion because of the small opening in the hymen. The vagina in these cases is in reality an abscess sac, and this condition calls for drainage, just as does any pus cavity. Under these circumstances administer chloroform and thoroughly dilate the vaginal orifice, wash out the vagina with a 1-1000 bichlorid solution, wipe it dry with cotton, and apply a 10 per cent. solution of argentic nitrate, or a 1 to 2 per cent. solution of protargol. The application is made best through a Kelly urethral speculum. After this slight operation the discharge will markedly decrease and the dilation of the parts will allow the passage of a soft rubber catheter or a small speculum with ease. Then applications of the above solutions can be applied without causing much pain, and douches of bichlorid or protargol may be administered daily. The treatment, however, must be persisted in until repeated microscopic examinations have shown that the vagina and urethra are entirely free from germs.—*American Medicine.*

---

### SURGERY OF THE HEART.

Not so very long ago a class of medical students listened with suppressed indignation to a lecture upon the methods of ligating branches of the coronary arteries. They felt that the subject was absurdly theoretical, and that a patient to whom such measures might be applied, needed the services of an undertaker rather than those of a surgeon. And, although these were unpracticed students, their opinion fairly reflected that of the mass of medical men, for the belief has been, and is still, more or less current, that any manipulation of the heart, not to speak of operation upon it, would induce fatal shock.

Undeterred, however, by this superstition, surgeons have not only ligated bleeding vessels upon the heart, but they have successfully sutured penetrating, incised wounds of the cardiac wall. The first intervention of the latter character was by Capellen, an Italian, in 1896. Since then, twenty-four cases have been thus operated upon, with eight recoveries. The first of the three operations in this country was done by H. L. Nietert, Superintendent of the St. Louis City Hospital, and was performed April 20, 1901. An abstract from the report of this case was published in these columns not long ago. George Tulley Vaughn has also reported a case. It has since been Nietert's fortune to meet with another case, in which operation resulted successfully, and which he reported before the Society of City Hospital Alumni at its recent meeting. The case was that of a negro, named Daniels, employed as a roustabout on the Mississippi river steamer, City of Chester. At Chester, Ill., the negro was stabbed by a companion. After the receipt of the wound, Daniels walked thirty or forty yards and fell unconscious. He was carried aboard the steamer and brought to St. Louis, where he was taken to the City Hospital, arriving there several hours after receiving the injury. Examination showed a stab wound near the left nipple, an increased area of cardiac dullness, and a splashing sound accompanying the heart movements. The thoracic wall was opened, making use of the osteoplastic flap, and upon exposing the heart it was found to have an incised wound about three centimeters in length, from which a quantity of blood was ejected at each heart-beat. The cut was through the wall of the left ventricle and in its long axis. Nietert closed the wound with two interrupted silk sutures. Drains were put in at the time of operation, but these were removed within a day or two. The patient was well on the road to recovery when an empyema developed. This was opened and drained in the usual manner. Recovery of the patient is now complete.

His experience with these two cases has stimulated Nietert to seek a method by which the heart sac may be entered without going through the pleura. This he has accomplished after a series of experiments upon cadavers, and he has devised an osteoplastic flap including part of the sternum and portions of the second and third ribs.

The work of this operator in this line is especially creditable, and it will undoubtedly elicit further valuable research into the technique of heart surgery.—*St. Louis Med. Review.*

---

### SURGICAL HINTS.

In spinal cocaine anesthesia the abolition of pain may be delayed for some time. It has been observed to come on as late as forty minutes after the injection.

If obliged to leave a catheter in the bladder, always avoid employing a metallic instrument. Their rigidity and their tendency to become rapidly incrustated render them soon harmful.

In burns of the mouth or throat, particularly in children, give frequent small doses of codliver oil, or sweet oil, with lime water. It will act both as a food, and, as a form of Carron oil, as a dressing.

Sugar placed in the water, or the use of simple syrup, will greatly facilitate the removal of plaster-of-paris from the hands.

after applying plaster dressings. The use of sweet oil is also serviceable for this purpose.

Whenever an aspirator is used, the operator should be careful to create only sufficient vacuum to cause the fluid to flow, since too complete a vacuum may draw the tissues together in such wise as to facilitate puncture of important parts, such, for instance, as in the case of a compressed or collapsed lung.

Surgeons have now quite generally abandoned the procedure of washing out the pleura after operations for empyema. Washing out does not seem to shorten the period of recovery, and it may, if repeated, cause a tendency to the establishment of a permanent fistula. It may also interfere with the formation of adhesions, thus preventing the union of opposite layers of the pleura.

In acute abdominal conditions, such as strangulation, obstruction or appendicitis, it is wise to withhold opium in order not to mask the symptoms and induce a false sense of security. But as soon as an operation has been decided upon, if there is to be any delay in its performance, it would be cruelty then to abstain from its use. It will relieve pain, thus diminishing shock, and will make the induction of anesthesia more easy.

When, after a laparotomy, there is evidence of intestinal atony, the bowels must be made to move. Purgatives, either mercurial or saline, may prove inefficient, and enemata must be resorted to. A high tube must be used. The most effective substances are the saturated soluted of Epsom salts, in quantities of a pint or more, or the mixture of Epsom and Glauber salts, each two ounces, with two drachms each of turpentine and dried oxgall, in a pint of water.—*International Journal of Surgery.*

---

### ANGIOMATA.

Angiomata, according to William Osler (*Bulletin of the Johns Hopkins Hospital,* November, 1901), are very peculiar and remarkable stuctures. Apart from the big nevi and angiomata with surgical relations there are :

1. The pin-point, punctiform, capillary angioma, of which few skins lack examples. They may be numerous, but they are rarely disfiguring. They appear and disappear. For ten years Osler had one the size of a pin's head on a finger.

2. The solid, nodular nevus, ranging from one to four or five millimeters in diameter, forming a definite little tumor, either sessile or pedunculated, and very common on the back.

3. The spider angioma, formed by (*a*) three or four dilated veins, which converge to and join a central vessel; or (*b*) which unites at a central bright red nodule projecting a little beyond the skin. They are very common, and doctors are often consulted about their presence on the face.

As examples may be found on the skin of nearly everybody, these three varieties may be regarded as almost normal structures.

When the punctiform or spider angiomata increase greatly in number they are greatly disfiguring. Angiomata have a curious relationship with affections of the liver. In cirrhosis, in cancer, in chronic jaundice from gall-stones, spider angiomata may appear on the face and other parts. They may be of the ordinary stellate variety, like the stars of Verheyen on the surface of the kidney, or the entire area of the star may become diffusely vascularized, so that there is a circular or ovoid territory of skin looking pink or purple, owing to the small dilated venules. A dozen or more of these may appear on the trunk, or even larger ones may appear. And lastly, in a few cases of disease of the liver Osler has seen large, mat-like telangiectases or angiomata involving an inch or two of skin, and looking like a very light birth-mark, but which had appeared during the illness. The skin was not uniformly occupied with the blood-vessels, but they were abundant enough on the deeper layers apparently to give a deep change in color, and to form very striking objects. The dilated venules on the nose, and the chaplet of dilated veins along the attachment of the diaphragm, are not infrequently accompaniments of the spider angiomata in cases of disease of the liver. The writer says that recently he has seen the spider angiomata appear in the face in a case of catarrhal jaundice.—*Medical Age.*

### NEEDLESS OPERATIONS.

It is instructive and refreshing to read an honest, frank statement of facts, such as were embodied in an article in *The Boston Medical and Surgical Journal* of January 16, 1902, by Dr. John C. Munro, of Boston, on "Needless Laparotomies, With a Report of Eight Cases."

"These cases were those in which no surgical lesion could be discovered at operation in spite of the fact that the symptoms, so far as they could be analyzed, led definitely to a diagnosis of some grave surgical trouble within the abdomen."

The surgeon says, further, that the diagnosis was open to criticism and suggestion, and was speculative by necessity. The eight cases include acute phosphorus poisoning, hypertrophic cirrhosis of the liver, two cases of nephritis, two cases of alcoholism (one with morphinism), a case of obesity, and one of supposed pancreatic cyst.

In some of the cases painstaking examinations were made, while in others it was purely routine under the existing circumstances, yet all were debated, and the exploration advised or undertaken after excluding many possible diseases. Such an array of internal diseases coming under the surgeon's knife offers many points for discussion, yet it is often the only method of arriving at a diagnosis, and perhaps it is better on the whole to know rather than to speculate on many of the chronic, misleading and undiagnosed forms of disease.

Many patients who occupy beds in the larger city hospitals are brought in suffering from such acute symptoms and with such meagre and unsatisfactory histories that relief is imperative, before a careful analysis of symptoms is possible. Often such cases are on the verge of fatal collapse, and the surgeon feels justified in operative exploration. In the case of hypertrophic cirrhosis and with the patient who had a history of gastritis and nephritis and the man with obesity, the exploration was done without harm; indeed, in the first two the operation was followed by speedy relief of all symptoms. The surgeon who trephines for possible brain tumor or other focal lesion, is frequently disappointed in his failure to locate the disease, and yet the patient makes a full and complete recovery from what seemed to be an advancing destructive process. Evidently, the same thing applies in abdominal work, as shown by the above report, and as often found to follow where only an ascites or a tubercular peritonitis is present.

In spite of such reports, the few cases that are benefited by a needless exploration are not to be compared with the many in which erroneous diagnoses are made and the patient subjected to needless or harmful surgery.

The paper of Dr. Munro is well worth reading, not only for the fearless and open report, but for his evident care in the study of his cases, his association with other men in his consultations, his admissions of error in diagnoses, and the difficulties which are thrown about the physician and surgeon in their efforts to ascertain actual conditions. Such an article is helpful in many ways, and illustrates anew the necessity of closer application between surgeon and internalist.—*Northwestern Lancet.*

---

### THE METRIC SYSTEM.

There has recently been, in Congress, a very energetic discussion regarding the adoption of the metric system with a view to making it the accepted system in weights and measures throughout the entire country. This agitation is not new, such discussions have been of frequent occurrence for years past. Teachers, especially those in chemistry, go to extremes in their demands for the adoption of this system, to the exclusion of all others. The writer has known teachers in chemistry who were such extremists, that having made their first question on an examination paper, one involving the metric system if such question were not answered correctly, the paper was thrown aside, and the student plucked in that department. It is true, that the system is simple, it is accurate, it is valuable, it is a time-saver, and yet for an excellent reason it will not, in this generation, if ever, become universally accepted. It is strange, indeed, that the reason referred to has not carried with it more weight among the ardent supporters of the system under discussion. We use the metric system in our money affairs, but we never talk it. Instead of referring to ten cents as one-tenth of a dollar, or five cents as one-twentieth of a dollar, we call five cents a nickle, ten cents a dime, and twenty-five cents a quarter of a dollar. Here is the key to the situation: The Anglo-Saxon mind from time immemorial has thought in halves, quarters and thirds, not in tenths, hundredths and thousandths, and no legislation in the universe, and no amount of rabid criticism will change the situation.

Let the publishers of text-books learn wisdom from the situation, and not break their financial heads against the stone-wall of opposition, which this law of thought presents, and which they are powerless to change, and, while printing their books it is eminently proper that they should incorporate the metric system for those who desire it, and for the advanced students who would be handicapped without it; let them not fail to accompany it with the equivalent values in the popular system, cumbersome though it may be. A reference to the prescription files of druggists the country over will demonstrate how very small has been the progress among physicions who, more than any other class of men, would be expected to appreciate the metric system at its true worth.—*Med. Herald.*

---

### THE HUMAN FORM DIVINE.

The fashion plates of the past half century form a museum of caricature that for grotesqueness no professional artist in that field will ever be able to equal.

It is well for the race that few real women even attempt to bring their figures to equal the ungraceful impossible burlesques on human anatomy that are every week perpetrated in the pages of the journals devoted to feminine fashions. To accomplish the extremes of Parisian fool-killing fashions would require each ambitious imitator to be anatomically dissected and resected. Just at present her fore-end, her nautical prow as she sails down Fifth Avenue or Broadway, needs to be thrust several feet forward of the rest of her body like the jibboom of any regular schooner or other craft. Her belt line is no longer horizontal but approaches the vertical, at least to the extent of an angle of forty-five degrees.

In the name of the Goddess of Health, and of the artistic shades of the Venus de Milo, in the name of their sainted grandmothers, what are the curled darlings who ape the outlandish, sense-insulting and art-shocking figures thinking about? Do they imagine that the vapid and brainless dudes who ogle them as they pass have any other feeling than one of derision and disgust?

And yet, semi-sensible women make freaks of themselves by half-way imitations of the idiotic dissections foisted on society by the Parisian man-milliners and the demimonde. If she rejects it for her own figure, mamma tolerates if she does not even indorse it for her ambitious daughters. Shame on women who have no more womanhood than to surrender to the senseless and distorted fads of the foreign fashion-mongers.—*D. & H. Gazette.*

---

### THE FRENCH COMMISSION ON TUBERCULOSIS.

The French Parliament, in a recent session, constituted a commission of thirty-two members to investigate the causes and prevalence of pulmonary tuberculosis in France and the progress that has been made toward its cure. Mr. Covert, the United States Consul at Lyons, France, has sent an abstract of the report of this commission to the State Department, which is published in Public Health Reports. The work of this commission is one of the recent exhaustive investigations of pulmonary tuberculosis and the conclusions are worth noting. In the first place, the commission finds that the disease is a real national peril to France, because the population is almost at a standstill. Furthermore, the disease seems to be on the increase both in the Army and in the Navy as well as among the civilian population. The great source of the spread of the disease is the dried sputum of tubercolous patients, although it may also be disseminated by the milk and perhaps by the meat of tuberculous animals. In this respect the recent pronouncement of Koch is, we think rightly, ignored.

Alcohol, overcrowding and overwork are predisposing causes of the disease. The commission recommends that people be prohibited from spitting on floors and upon the street. In this matter the French are behind us, and it is gratifying to read that America is considered an example in regard to this sanitary measure. The open-air treatment is advocated, and the report declares that by this means consumption is curable. The construction of sanatoria for consumptives is recommended. The commission believes that the children of consumptives, by the mere fact of their birth in a state of organic weakness, are predisposed to the bacilli. This raises a point, recently made by Flick, that the children of tuberculous parents are to a certain extent immune to tuberculous infection; a subject upon which a special study would be of great value.—*The Philadelphia Medical Journal.*

---

### DUHRING'S DISEASE IN CHILDHOOD (GOTTHEIL).

(Author's Abstract.)

Dermatitis Herpetiformis, first described by Professor Duhring, of Philadelphia, is probably of commoner occurrence than is generally supposed, more especially in children; two cases are described by William S. Gottheil of New York, in the June number of the *Archives of Pediatrics.* The resemblance at first sight to an ordinary eczema, dermatitis, or impetigo is marked, and doubtless cases of the disease are not infrequently so classified. The points which distinguish the less common affection are:

1. The extreme obstinacy and chronicity of the malady; it being prolonged almost indefinitely by successive exascerbations or relapses.
2. Its original herpetic character and subsequent multiformity of lesion.
3. The intense pruritus.
4. Its recalcitrancy to treatment.

Any apparent eczema, dermatitis, or impetigo in children presenting these features should be carefully observed; a certain number of them will undoubtedly be found to be cases of Duhring's disease.

---

### ACTINOTHERAPY (GOTTHEIL).

(Author's Abstract.)

In a preliminary communication upon the use of concentrated light in the treatment of dermal affections, W. S. Gottheil briefly reviews the work done by Finsen, Kine and others in this field, and describes the arc light that he employs for the purpose. This is at present the only available source for the actinic rays of sufficient volume and intensity for therapeutic employment. Sunlight is of course the best, and is costless; but it is too uncertain for satisfactory use. No combination of incandescent bulbs, run on the ordinary continuous or alternating commercial current, is sufficiently actinic, and the apparatuses arranged with them practically give us heat and no light baths.

The author employs an apparatus called the Actinolyte, made by Kliegl Bros., of New York, which can be adopted to either the continuous or the alternating current, uses from 25 to 55 amperes and gives a concentrated circle of light of from 20,000 to 30,000 candle power. He is not prepared yet to publish his results; but the progress of cases of lupoid and syphilitic ulceration has been most encouraging. The cosmetic results of this non-operative and painless method of treatment are especially good; a point of the greatest importance, of course, when the face is involved.—*The Medical News, July 6, 1901.*

---

### REMOVAL OF GUNPOWDER STAINS.

BY DR. E. G. CORBETT, HAMPTON, FLA.

(Published by *The Medical World* of Philadelphia, Pa., February, 1902.)

On Christmas Day a boy of twelve filled a vaselin bottle with powder and exploded the same. I arrived on the scene about three hours after the accident and found the cornea and sclerotic of both eyes and the face literally blown full of powder. I removed a dozen or more flakes of powder from each cornea with a foreign spud; also removed the powder from the sclerotic. Did the operation under a four per cent. solution of cocain. After the operation I used a fifteen per cent. solution of Hydrozone in the eyes. After removing the particles of glass from the face, I kept a cloth over it saturated with a fifty per cent. solution of Hydrozone. At the end of two weeks I used a saturated solution of boric acid in the eyes and painted the face twice daily with equal parts of Hydrozone and glycerin. The eyes are well and powder stains have disappeared from the face.

